# Consumption

**Published:** 1856-09

**Authors:** 


### HALL'S JOURNAL OF HEALTH.

OUR LEGITIMATE SCOPE IS ALMOST BOUNDLESS: FOR WHATEVER BEGETS PLEASURABLE AND HARMLESS FEELINGS, PROMOTES HEALTH; AND WHATEVER INDUCES DISAGREEABLE SENSATIONS, ENGENDERS DISEASE.

VOL. III.] SEPTEMBER, 1856. [NO. IX.

### CONSUMPTION

Is a gradual destruction of the lungs, a slow wasting away of the "lights," as they are called, by many, when applied to animals.

There are various kinds of consumption, Consumption of the Throat, Consumption of the Bowels, but when the word "Consumption" is employed, by the great mass of people, it means Consumption of the Lungs, and there arises in the mind the idea of cough, of pale face, of wasted flesh, of stooping frame, of slow and careful walk, of large round eyes, the white predominating, a waxen countenance, as serious as the grave, with a general look of anxiety and distress, which wakes up warmest sympathies in hearts that seldom feel at all.

The reason of this universal application of the word "Consumption" to the lungs is, that so many are destroyed by it in civilized society. It is estimated that one adult out of every six, dies of this disease. Such being the case, scarcely a man who reads these pages, but will, sooner or later, even if he escape himself, have his eye moistened or his heart stricken by the work of this great destroyer. These things being so, every man owes it to himself, to his family, and to his kindred, to obtain a knowledge of this disease, as to its nature, its causes, its prevention, and its alleviation or cure. Information of this kind can be communicated without the necessity of long disquisitions, of tedious investigations and distressing niceties of discrimination. The ailment is so common, it is of such every day occurrence, that most readers are familiar with it, can pronounce upon its existence in the person of another with considerable correctness, in its decided stages; yet such is the deceptive character of the malady, that it is almost a symptom of it, that the man himself cannot be made to believe in its presence, in his own person, until within the last weeks of his existence, and in very many instances, not until the last, the very last hour of conscious life. On being called to a gentleman on one occasion for the first time, it was apparent that he would soon die. When informed of his true condition, he replied, "Doctor! you do not understand my case; if I only had a carriage to ride about the city, I would be a new man in a few days." He died that night. Another was a young gentleman of high promise. I had been attending him for some time and steadily acquainted him with the progress of his disease. But he constantly talked of his plans and purposes, with that patronizing consciousness of the groundlessness of my fears, which it was difficult to withstand with equanimity. "Why," said he, "my mind is as clear as a bell." And so it continued to be, on all other subjects. Soon after, his factor came to render an account of bills of sale of his cotton crop. He examined it with great care, and in adding up the column, detected an error of a few dollars. He died the next day.

The great reason of this deception is, there is sometimes no pain at all, no suffering, no apparent violence, and the patient proposes to himself the question, "How can I be seriously ill, when I am conscious of no distress?" He feels that if he only had a little more strength, he would be well enough. Besides, there are moments during any day either soon after a sound sleep, or in the excitement of fever, when he feels as if he had that strength, and this increases the illusion. A young gentleman of family and fortune was travelling homeward with this disease upon him. On waking up early one morning, he said to me, "I feel as if I could travel a thousand miles." The same week, he slept the sleep which knows no waking.

There is something fearful in the thought of being a victim to such a delusion; of travelling along the very verge of the grave, believing ourselves to be treading on solid ground, all unconscious of the actual fact, that every moment it is crumbling from beneath us.

There is a moral reason for this strange delusion. We are all loth to admit unpleasant truths. A man in business is the very last one to perceive that he is a broken merchant. His neighbors have known it long ago, but he himself does not be-become fully conscious of the fact, until the sheriff turns the key on his door.

One of the consequences of this delusion is, that it prevents the person who is the subject of it, from taking those active measures which would avail to defer the malady indefinitely, if not to accomplish a permanent cure. Forewarned is to be forearmed. A stitch in time here, saves a million.

As the reader, however strong and robust now, however high in health and buoyant in hope of years, long and successful, may at any time become the subject of a malady so deceptive, he will, if he is wise, be at pains to obtain such a knowledge of it, as to prevent him becoming a victim to its delusions.

There is another thought in the minds of men, in reference to this affection, which is not less illusory than the one already named. Persons often express themselves thus, "I wish I could die of consumption, it is so painless a disease, and gives one full time and fair warning to prepare for death." The time it does give, as about two years is the average of its duration. As to the warning, it is certainly given in tones loud enough to be heard by thousands afar off, but not loud enough for the ears of the man himself--given in arguments so convincing and so palpable, that the humblest intellect can perceive them, but not clear enough to make the invalid himself appreciate their power.

As to the painless nature of consumption, the delusion is as complete as it is general. In some very few cases, there is comparatively little pain, one in a million perhaps. In all, there are times of comparative exemption from severe suffering. But the very countenance of a consumptive shows an abiding distress, so continued, so ever present, that it has fixed its unmistakable imprint on the whole man. "Death by *the drop*" as it is called, where a single drop of water falls upon the head at one spot, is said to be rather pleasant at first, but continued hour after hour, day and night, soon produces delirium, and if continued, the man becomes a raving maniac for life. But there is nothing in consumption which is even transiently agreeable, not one symptom, but many. The whole man is diseased, every drop of his blood is on fire. The ceaseless fever burns out his life. And when all his fat and flesh are consumed and there is no more oil to feed the flame, no more carbon to keep up the dying fires, nothing left but skin and bone and tendon and ligament and strings, then he begins to freeze. The fingers first, and feet, all his efforts cannot keep them warm. Week after week the cold chill of death creeps higher and higher, nearer and nearer in the slow progress of months, until the heart itself becomes an icicle, and the man is no more.

So far from death by consumption being an easy one, there are few maladies which involve a more fearful amount of suffering in the aggregate. The shivering chill of the forenoon, the burning fever in the after part of the day, then the drenching night sweat, clammy and cold as death, and thus for days and nights, for weeks and months, if there is any "*ease*" here, we cannot bring our mind to perceive its reality.

### THE COUGH.

The very sound of it, in an advanced stage of the disease, is unspeakably distressing. At night fall, the poor, wasted, wearied body longs for repose, the eye looks longingly to the bed, while the effort for undressing seems herculean and the time requisite for it, an age. The fleshless skeleton totters to its pillow, and on the instant, the very instant, the cough begins, at first hard and dry; nothing comes up. Cough, cough, cough! racking, jarring, straining. He feels "If I could only get it up, how sweetly could I rest." And he coughs on. The slow minutes are hours, and the hours, ages, as he tosses on his bed, the wan face bathed in the perspiration of exhaustion, or flushed with the fever which is burning out his life. At last a mouthful does come, and he hopes for rest. A mouthful of lungs rotted away, falling upon the floor in thick yellow lumps, with spraggling, ragged edges, giving the coveted repose, not for hours, nor even minutes always, but for one, a few brief seconds only, and then begins again the sad, sad labor, to be completed only until the grey of the morning comes, when more dead than alive, and from utter exhaustion, the patient falls into a troubled sleep, as unsatisfying as it is brief; and more weary than when he retired, he leaves the bed with the same confident hope of relief, as he had on retiring, and as certainly to be unrealized; and thus baffled from sunrise until evening, and from nightfall until the morning comes, he wears his life away.

Death by consumption easy! Look at it. The appetite is usually good, he looks forward to the eating hour with interest and satisfaction; he thinks over and over again how he would enjoy this and that article of food, and in the delirium of anticipation, he projects himself into the long years of the future, and revels in thoughts of how, when he gets well again, he will take care of his health and purchase him a little farm, and ambitionless of society, and position, and equipage and office, and wealth and a name, he will devote himself to the leisure cultivation of fruits and flowers, and feast day after day on pure milk and fresh eggs, and new butter, with vegetables from his own garden and honey from his own hive. Upon this elysian reverie the call to dinner breaks, and with watering mouth and eager expectancy, forgetful of every symptom, oblivious of every pain and suffering, he lays himself out for a hearty meal. He eats much and long, and enjoys it. Food never tasted half so good and he rests not until the feeling of perfect satisfaction comes over him. But the first material change of position, moves also the fluid mass of rotted lungs within him as certainly as the motion of a glass changes the position of the water in it; this change of matter to a fresh part of the lungs, the sensibilities of which have not been obtunded by the long pressure of this decayed substance on one spot, excites a tickling sensation, not in the lungs themselves, but in the hollow at the bottom of the neck in front, just as the eye sees, not at the eye ball, but on the retina, just as the stricken elbow gives the sensation at the distant finger ends, this tickling gives cough, a mere heck at first, but each successive heck causing another quicker and more decided, until a regular hard cough sets in, bringing on gagging, and soon the whole meal is cast up, for no rest comes until it is all brought away. And thus it is with every meal, for many of the last weeks of life, and in which we look around in vain for any "*ease*."

To listen to the merry laugh of others, but no such mirth to you, for it brings on a cough, which may last for the next half hour. You hear the song of gladness in others, but the first note you strike, brings on the inevitable cough. You hear some splendid speech, or contemplating some noble action, or gazing at some magnificent object of nature or of art, the thrill of admiration sweeps over you! and the hated cough comes on by the very emotions of the mind.

You look out upon the gay fields of a summer's morning, or upon the bustling crowd in the business street, or the more joyous promenaders of the avenue, or the sleigh bells tingle by on the bed of driven snow, and the ceaseless laugh, or the loud yell of youthful recklessness, all, all pass before you with sweet remembrances, the sweeter from the distant impression, that none of these may be ever yours again. In none of these can you participate now. There is no strength of limb to walk the summer fields; there is not breath enough to enable you to keep pace with the busy crowd, no heart to join with the gayer throng, while the very thought of sleighing over the cold snow, causes you to shrink back with a shiver, and the sympathetic cold chill drives you from the window to the fire place. If there is any "*ease*" in aught like this, it is imperceptible to me.

But when confinement to the bed gives loud note of death, and one by one your delusions have all passed away, and you sit propt up by pillows, your only apparent enemy being the phlegm, which you wish to get away, there is less prospect of ease than ever. Every breath you draw makes it boil up and rattle and flutter within you. You feel as if a little cough would bring it up. But the sensibilities of the parts are in the main taken away, for you are dying. You have not strength to cough, except at intervals, and then so faint, that it does not "*reach it*," or if it does, it barely brings it up to the throat, when it falls into the "Swallow," and goes down into the stomach, there to be mixed up with your food and drink, whole pints of it in a day sometimes! O let me run away to some distant planet, to escape so horrible an end.

At last there is not strength enough to bring it as far up as the gullet, and accumulating every hour, the remaining lungs become clogged up, the slightest amount of air gets in, and a dreadful oppression comes over you; you feel as if one good, long, full breath would be perfect happiness, and no giant could labor harder to get that breath than you. In that terrible effort, the effort for life, the eyes become glary, the mouth remains open, the bosom heaves laboriously, each partial breath a groan, large drops of clammy sweat stand upon the forehead, the speechless tongue, the pulseless wrist, the fading light, and all is over!

### CAUSES OF CONSUMPTION.

Such being a history of the progress and end of this ruthless disease, it may be instructive to inquire into its causes.

Suppose we close the books, lock up the libraries, consign all theories to the grave and rely upon that best of all informers, *observation*, and with the aid of common sense, endeavor to learn some facts for ourselves and deduce conclusions, which it is impossible to gainsay.

The first idea which strikes us, on mention of the word "Consumption," is that of a pale, emaciated form. We all know that paleness of the face arises from the absence of the natural amount of blood, the pure blood of health. Emaciation forces on the mind the conviction of a want of nourishment. We then arrive at that most important fact, underlying all others, that the essential nature of consumption is a marked deficiency of flesh and blood, paleness and emaciation being its universal attendants, conditions, or symptoms, without which it never can exist. It must then strike the thoughtful reader, that if paleness and emaciation are always present in consumption, debility must be as inseparable from it, as death is inseparable from the grave; and this other conclusion is equally obvious, that inasmuch as *Paleness*, *Emaciation* and *Debility* are always present in consumption, that whatever causes paleness, emaciation and debility, in continuance, is capable of causing consumption.

It must not be inferred here, that every man who is pale, emaciated and weak, has consumption. The fact is stated, and there left, "*Paleness, Emaciation and Debility are never absent in any case of common consumption of the lungs, and that whatever causes these, in permanence, is capable of causing consumption.*"

Now, instead of going on naturally, and stating the causes of consumption, we will first proceed to show what are *not* the causes of consumption, in order to make the contrast more instructive and impressive.

A man naturally shrinks from taking ground antagonistic to generally received opinions and it ought never to be done, except on mature investigation, on the clearest conviction and with the fullest impression, that it is for the public good, by advancing the truth. It is by the pure truth that the world is to be millenialized, and made a paradise; and the universal sentiment should be, *Let truth prevail, wherever it may lead.* We have nothing to do with the consequences of pure truth. He who is Truth itself, will take care of that.

"*Tight lacing,*" as it is called, does not originate consumption; its tendencies are to prevent it, if not actually present, and to cure it, if it is.

All physicians know that consumption attacks the top of the lungs, under the collar bone, and that long before it reaches half way down, the man dies, not actually for want of enough sound lungs to live upon, for persons have lived to a good old age, who have had but one half of the whole lungs in healthful operation, but they die from the effect which the disease has had on the whole system.

*Tight Lacing* affects the lower portion of the lungs mainly, and causes the person to breathe less with the bottom of the lungs and more with the top. We have seen that the bottom of the lungs can take care of themselves. It is not one time in many thousands, of those who die of this disease, that the lower portions are materially affected, if at all.

The reason that the lower portion of the lungs is the last to become consumptive is, that it has more room for full action, the lower portion of the ribs and the stomach are distensible, and in drawing a full breath, we see how readily they swell out. And consumption never can exist where the lungs have free, full play to the influences of a pure atmosphere; and even when the atmosphere is foul, those portions which work most freely, are the last to become diseased; and conversely, the upper parts of the lungs, being encased by unyielding bony walls, have not the capabilities of distension which the lower portions have, and consequently are more liable to disease.

It is intuitive to us all, that those who are out of doors most, who run and race about most, who are most active in their pursuits, are less liable to consumption than those who follow still occupations, indoors. Reasoning from a general fact we would conclude then, that very many more women die of consumption than men. But it is simply not so. Now what is the reason? Women breathe more with the upper portion of the lungs than men do; any one's observation will confirm this assertion. Therefore, the province of woman being more naturally within doors, a beneficent Providence seems to have so created them, that there should be an antagonism within them, and beyond their control, to the otherwise natural liabilities to the disease. We therefore arrive at the inevitable conclusion, that compression of the lower portion of the lungs, throwing as it does, a large part of the breathing and distension to the upper portion, does thereby render the upper portion less susceptible to disease. We mean moderate compression.

What then becomes of the impression that *tight lacing* originates consumption? It must simply go the way of multitudes of specious errors.

The reader will please bear in mind, that we do not advocate tight lacing. On the contrary, we are opposed to all kinds of compression, all impediments to the fullest and freest action of every member and portion of the human body, that there should not be a buckle or button, or string or pin or pad about us, more than is absolutely necessary to keep our clothing from falling off our bodies. We are only speaking of *Tight Lacing* in its bearing on consumptive disease. If the statements which we have made are startling to some, and inconclusive to others, let us appeal to facts. The advent of the *Cold Water Era* has been the means of introducing many wholesome truths. Its friends have been energetic, enthusiastic men, not over bright, it is true, but they have been sincere; whether they have done more good than evil, it is not now necessary to inquire. But one effect, which their efforts have aided very considerably in bringing about, is the comparative abolition of tight lacing, and for their labor they deserve much praise, showing as it does, that they are not so bigoted that they cannot *follow!* in the path of *educated* medicine, when they believe that path is truth.

It has taken ten years to bring about the abandonment of the corset. And now we have two simple questions to propose.

Do fewer women die of consumption to-day, when the corset is in comparative desuetude, than ten or twenty years ago when tight lacing was all the rage? All statistics show that there is no remarkable change.

The people of the town are more dressy than those of the country, more apt to go to extremes, and more universally follow leaders. Is the proportion of women who die in town of consumption, materially greater than in the country? Statistics say no. *Binguet* says of ninety-one women dying of consumption, forty-seven were brought up in town and forty-one in the country, showing a difference of only one-seventh in favor of the country. But women wear corsets and men do not, yet in 1837, of persons dying in a Paris Hospital of consumption during four years, one-tenth more were males than females.

In England, the returns of the Register General show, for 1845, that in the country, where corsets are less worn, more women die of consumption than men; but that in London and other large cities, the mortality from this disease is much less among women than among men. Now it is reasonable to infer, that there is less tight lacing among a farming population than in a city, and the above fact shows that where tight lacing most abounds, consumption is less prevalent. We do not say that tight lacing has the credit of this exemption, but it is clear that if tight lacing does tend to produce consumption, there are causes in operation which greatly overpower that tendency; hence we have some reason to infer that such a tendency has no appreciable existence.

It is thus seen, that in cities, where corsets are more worn, fewer women who wear them die of consumption than men, who do not wear, notwithstanding their greater liability to the disease from their sedentary indoor employment, and so great is the difference of liability, as to in and out door occupation, that in Geneva, thirty-seven per cent. of varnish painters died of consumption, while of gardeners who perished by the same disease, there was only four per cent. Of painters, tailors, engravers, clerks, &c., a hundred and forty-one out of every thousand died of consumption, while only eighty-nine of agriculturists, blacksmiths, slaters and the like died of it. With these strong facts before us, we are obliged to infer, that there is something in woman which is exemptive of consumption, and it is legitimate to conclude, that one of the elements of that exemption is a fuller, freer working of the upper portion of the lungs, which is uniformly the seat of the disease. This is fully coincident of the admitted fact, that full, free breathing, tends to prevent consumption. If additional proof of this most important practical fact is needed, it is found in the uniform statement of great travellers and close observers. Buffon writes that all animals inhabiting high altitudes have larger lungs, and more capacious chests than those which live in the valleys. Wilson and Audubon agree that birds which practice the highest flights have the largest receptacles for air. Thus it is, that reasoning from birds and animals to men, there is no city in the world so free from consumption as Mexico, it being nine thousand feet above the level of the sea. For in the same year, while three persons out of every hundred died of consumption in that city, there perished by that same disease, in our larger cities, eighteen persons out of every hundred. Why? Because the rarified atmosphere of high altitudes, compels the breathing of larger volumes of air, to answer the wants of the system, there being less substance in a rarified, than in a condensed atmosphere; and this taking in an increased volume of air at every breath, produces a corresponding development, distension of the lungs, which is, as we purpose to show hereafter, the fundamental essential, in the prevention, the amelioration, the cure, in every case of consumption ever reported.

HEREDITARY TENDENCY *is not specially promotive of consumption; it is more nearly a preventive.*

To concentrate the argument in a few words, and make the ordinary, the every day observations of reflecting men constitute the proof, it is only necessary to draw attention to one familiar fact. *It is not the feeble of adult life who soonest die.* We can all bring multitudes of cases to our remembrance, where the stout and robust and strong, full of vigor and health, have long since been laid under the clods of the valley, and whose names are remembered to the very few; while of others, so tottering and frail, that no one believed they could possibly live beyond a few short years, an age or two have passed away, and they are living yet, and likely to live a good long time to come. At the age of twenty-two, P. S. was believed to be in a hopeless decline, "she can't possibly live beyond a year or two," was a very common expression among her friends. But she did live, has survived three husbands, and half a century besides. And this day, we know her to be in better health than at any time within the last ten years, and bids fair to reach "four score."

The explanation of this fact is simple, conclusive, and of great practical value.

The feeble feel the absolute necessity of taking care of themselves. They know that upon it hangs the question of enjoyment and suffering, of life and death; indiscretions, imprudences, tell upon their feeble frames, with almost telegraphic rapidity, and there is only one alternative, *carefulness or suffering.*

On the other hand, those who abound in vigorous health, feel that *their* constitutions are impregnable, that nothing can hurt *them.* Thus they are habitually negligent, careless, and often even reckless. The result is, they soon pass away, many of them long before their prime. Hence, practically, persons hereditarily consumptive, do not very necessarily suffer more from consumptive disease, than those who are exempt from this tendency.

This at least is the theoretical statement; but mere theory should never override carefully ascertained statistics. And to this very point, the attention of scientific men has been long drawn, and we only record the statement of one of them, and he had large opportunities of long and wide observation. "Hereditary predisposition to consumption is as frequent among persons brought up in the country as among those brought up in town. Those born of consumptive parents seemed not to be more liable to take cold than others."

The same writer states, that "of ninety-eight persons who died of consumption, thirty-three were naturally of a robust constitution, and twenty-one were of a feeble constitution.

*Bad colds do not originate consumption.* Truth is useful everywhere. Its practical application in physics and morals tends to ameliorate the evils of life and elevate our natures. Hence, we make the above statement, which many will consider as extravagant as it is untrue.

The result of the very prevalent opinion that bad colds beget, generate, originate consumption, is that, for fear of taking cold, many are induced to avoid going out of doors, except in the mildest weather; this causes them to remain in-doors, especially if invalids, full nine-tenths of their time, in this climate; hence, nine-tenths of their time they are breathing a vitiated atmosphere, which is quite competent to generate general disease where it is not, and aggravate what already exists.

But suppose the impression was as general that bad colds were curative of consumption, as that they originated the disease, then, the consumptive would expose himself more freely, would go out in all weathers, hot or cold, rain or shine, fair or foul, burning or freezing, and with a kind of desperate recklessness, he would court what is now considered the danger, AND THAT WOULD CURE HIM!!

A bad cold can no more originate tubercular consumption, than powder could ignite without fire. When tubercles are already existing in the lungs, bad colds may develope them. As the powder must be there, before the fire can produce explosion, so tubercles must be in the lungs, before a bad cold can develope them into consumption, and the prevalence of tubercles is the result of operations going on in the system for years; while a bad cold has nothing in it which tends to produce tubercles, for it runs its course usually in ten days, just as measles run their course, or mumps, and then passes out of the system.

The reason of the prevalent belief of the connection between a common cold and consumption is, that cough is the distinguishing feature of both. Hence, whenever a consumptive gets worse, the almost invariable expression is: "I must have taken cold in some way, and yet I do not see how it can be so, for I have taken every precaution." So that, whenever the cough becomes worse, is more decided or troublesome, the invalid's inference is that he has taken a fresh cold. This is a delusion, and in its practical bearings, is a fatal one, as it results in more continued confinement to the house, in order to prevent taking cold, and to the securement of an even temperature, when, in reality, an even temperature, a temperature of room regulated to a degree for months together, is as certainly fatal in any case of decided consumptive disease as we can readily imagine. In the reading of an age, we do not remember to have seen a single case described in medical publications, in which a regulated temperature did not end in death. So, the fear of taking cold, in the belief that such a cold aggravates consumption, effectually cuts off the invalid from the most important of all means of cure. For, *without a full and free exposure to out door air, regardless of all weathers, no case of consumption ever has been cured; while with it,* AND IT ALONE, *many cases may.* Let the reader manufacture his own statistics on this point in this way. One person out of every six dies of consumption.

Of these, five have had bad colds a thousand times during their life, and here we have five thousand bad colds without a single case of consumption; and as to the man himself, he had a bad cold five, six or eight hundred times before, and under it all, he never became consumptive. And because one bad cold out of five or six thousand was reputed to have been followed by consumption, it is the slimmest of all arguments to make it the foundation of a conclusion, that consumption is originated in a bad cold. No theory ever worth a thought could stand upon a foundation like this, and since that theory originates a very general and practical and fatal error, we owe it to ourselves, every lover of truth, every humane man owes it to himself, to give the subject a stern and thorough investigation.

If, then, *Tight Lacing* does not originate consumption;

If *Hereditary Tendencies* do not practically make persons more liable to die of consumptive disease;

If *Bad Colds* do not originate consumption;

What are some of the more prominent and pregnant causes of a disease, under which there are suffering in England and Wales, every year, no less than seventy thousand human beings, and, no doubt, an equal number in the United States?

We have already seen that *Paleness, Emaciation and Debility* are symptoms which are always present in common consumption of the lungs; and, although these are not always indicative of the presence of consumption, yet it is a legitimate inference that, whatever causes these, is a sufficient cause for consumption; and, consequently, it is our duty to know the occupations, and callings, and pursuits, the intemperate prosecution of which inevitably induces, if persevered in, paleness, emaciation and debility. Let it be remembered, it is not designed to advocate the total abandonment of these pursuits, for they are useful and necessary; but to follow them only so far as they do not seriously impair the health. We know of no calling of human life, which may not be pursued with impunity, which may not be pursued in such a way as to promote health, if done judiciously, wisely, moderately.

What, then, are some of the callings of human life which, in our own observation, give the pale face, the wasted flesh and the feeble walk?

Indoor employments, especially those which do not demand activity on the feet, supply much the largest number of victims to consumption, while those who are out of doors a great deal are almost wholly exempt, or if attacked at all, it is the result of a change of life, to an inactive or indoor employment, or to some unpardonable instance of thoughtless indifference, or some hardy reckleseness.

Out of every hundred varnish painters, thirty-seven die of consumption. They live mostly indoors.

| | | | |
|---|---|---|---|
| Varnish Painters, | 37 | Slaters, | 9 |
| Tailors, | 14 | Agriculturists, | 9 |
| Engravers, | 14 | Butchers, | 7 |
| Printers, | 14 | Tanners, | 7 |
| Clerks, | 14 | Candle Makers, | 7 |
| Polishers, | 12 | Easy Circumstances, | 5 |
| Plasterers, | 12 | Butchers, | 5 |
| Sculptors, | 12 | Dyers, | 5 |
| Stone Cutters, | 12 | Bleechers, | 5 |
| Watch Hand Makers, | 12 | Watermen, | 5 |
| Carpenters, | 9 | Gardeners, | 4 |
| Blacksmiths, | 9 | | |

The influence which out door activities have on the general health accords with that had on consumption.

The average life of

| | | | | | |
|---|---|---|---|---|---|
| Stone Cutters is | 34 | years. | Surgeons, | 54 | years. |
| Sculptors | 36 | " | Masons, | 55 | " |
| Millers, | 42 | " | Gardeners, | 60 | " |
| Painters, | 44 | " | Merchants, | 62 | " |
| Carpeters, | 46 | " | Clergy (Protestant) | 63 | " |
| Butchers, | 53 | " | Magistrates, | 69 | " |
| Lawyers, | 51 | " | | | |

By a careful examination and comparison of these tables, which are regarded as merely approximative, it will be seen that there is a striking correspondence between the causes of general disease and the causes of consumption; that persons who are out of doors most, and most active, live longest, and are most exempt from consumption.

In speaking of the causes of consumption it is useful to remark, that among those who are least liable to consumption are persons in "*easy circumstances.*" What a loud and impressive lesson is here read to humanity. What a strong reproof to the men and women who are working their very eyes out for gold; who day and night, summer and winter, are tugging, and striving, agonizing after money, who rob themselves of necessary sleep, who stint themselves of necessary food and clothing and comfort, to hoard up that which *perisheth with the using*, who work beyond their strength every day of their lives in their struggle after the greed of earth. These are people of *uneasy* circumstances, and it is not they who are exempt from consumption, but those who are in easy circumstances, and being content there to remain, are in *easy circumstances* still. *To be in moderate circumstances, and take the world easy, that is the true philosophy of life.*

What a sad tale, that item about "*easy circumstances,*" tells of poor humanity! while they are almost exemptive of consumption, how forcibly does it speak to us of the converse as a cause. The uncertainty of to-morrow's bread! to not know where the next "*rent*" is to come from! to not know but in another twenty-four hours, one's family will be roofless! To lean day by day on the dagger of unrequited love, of misplaced affection, of confidence forfeited, of heart broken! To pine away in desertion, in hopelessness, in the consciousness that our life time has been a failure, and that it is too late to try again; to be young and all one's kindred gone, sister, brother, father, mother, all passed away; to be yearning for something to love and lean upon, but to meet indifference and coldness and rebuffs; or to be old, the sad and sole survivor of a large kindred, the friends of our school time, the associates of our youth, the companions of riper years, the dear, dear children of our prime, of these not one left, departed all—not "*easy*" circumstances these, but terrible; and no wonder, is it, that under them, the heart and body too, pine away, and only find an end in the consumptive's grave.

We then have arrived at a great fact that depressing mental influences are a "*cause*" of consumption, while in connection with it the interesting and instructive truth presents itself, that while moderate bodily exertion out of door exempts from consumption, immoderate labor or comparatively inactive out door employment invites the disease. The sculptor, who stands at his stone, chisel in hand, in the self-same square yard for days and weeks together, and for hours at a time in the self-same, almost immovable stooping position, is one third more liable to consumption than the agriculturist, who is constantly changing the position of his body, constantly bringing a large variety of muscles into exercise, and whose locomotion amounts to miles asunder every day. Nor is it less curious to observe that the gardener is one hundred per cent. less liable to consumption than the agriculturist; a sufficient explanation lies in the fact that his labor is more moderate, and uniform, attended with less anxiety and surrounded with the more pleasing associations which gather around fruits and flowers. The tastes of the man are compelled into exercise and his mind is drawn out, dozens of times every day in comparisons as to proportions, adaptations, appropriateness, and beauty, all pleasurable, all elevating, while the farmer's heart is eaten out by the two great cormorants, Season and Price. Did any man ever know a farmer who was not an habitual grumbler, who was not always ready with a too dry or too wet, too backward or too forward, too hot or too cold? We ourselves have known some, not many, who were habitually and humbly thankful for whatever kind of weather a kind Providence thought proper to send.

Whatever renders the blood impure tends to originate consumption. Whatever makes the air impure makes the blood impure. It is the air we breathe which purifies the blood. And as, if the water we use to wash our clothing is dirty, it is impossible to wash the clothing clean, so if the air we breathe is impure, it is impossible for it to abstract the impurities from the blood.

What then are some of the more prominent things which render the air impure? It is the nature of still water to become impure. It is the nature of still air to become impure. Running water purifies itself. Air in motion, drafts of air, are self-purifyers. Thus it is that the air of a close room becomes impure inevitably. Thus it is that close rooms bring consumption to countless thousands. Hence all rooms should be so constructed as to have a constant draft of air passing through them. The neglect of it, murders myriads. A man of ordinary size renders a hogshead of air unfit for breathing, consumes its blood-purifying quality every hour, so perfectly, that if a man could re-breathe a full breath of his own the next instant after its expiration without any intermixture with the outer air, he would be instantly suffocated. Hence sleeping in close rooms even though alone, or sitting for a very short time in a crowded vehicle or among a large assembly is perfectly corrupting to the blood. Close bed rooms make the grave of multitudes.

Among other causes of consumption are insufficient food or clothing; sleeping in basements or sitting habitually in damp apartments. A dog will become consumptive in a few weeks if confined in a damp cellar, especially if it be a dark one.

Hence the room which we occupy for the largest portion of each twenty-four hours should be the lighest, dryest, most airy and cheerful in the whole building.

As occasional causes of consumptive disease, there may be mentioned all suppressions, the sudden driving in of all eruptions, such as measles, tetter and the like, the sudden healing up of sores which have been running for a long time, without intelligent medical advice, in carrying off the drains of the system in another direction. Many lives are thrown away by ignorant officiousness, in applications to old sores: they are elated to the highest degree in having "cured up" an ulcer, which the "regular doctors" had failed to do after months of effort, but they fail to note the after fact, that within a very short time the "cured up sore" has broken out again, or falling on the lungs, has laid the victim in the grave.

It is the province of the skilful physician to know when to let alone as well as when to act. To do little or nothing is sometimes the highest wisdom.

